# Modeling the melting temperature of metallic nanocrystals: dependencies on size, dimensionality, and composition

**DOI:** 10.1039/d5ra01939g

**Published:** 2025-05-07

**Authors:** Yanli Ma, Ming Li, Haiming Lu

**Affiliations:** a Anhui Province Industrial Generic Technology Research Center for Alumics Materials, Huaibei Normal University Huaibei 235000 China liming2010@chnu.edu.cn; b National Laboratory of Solid State Microstructures, Collaborative Innovation Center of Advanced Microstructures, College of Engineering and Applied Sciences, Nanjing University Nanjing Jiangsu 210093 China haimlu@nju.edu.cn

## Abstract

The melting temperature is an extremely important property in describing the stability of metallic nanocrystals and can be modulated by the size, dimensionality, and composition. In this study, a new model was developed to comprehend these effects on the melting temperature by considering the surface stress and the size-dependent surface energy. The developed model predicts a decrease in the melting temperature with decreasing size or dimensionality. Moreover, for nanoalloys with identical size and dimensionality, the model suggests that the melting temperature decreases as the component with lower surface energy increases. Importantly, our model's predictions are consistent with experimental and simulation data, validating its accuracy and universality.

## Abstract 2

The depression of *T*_m_(*D*) with decreasing *D* results from the synergistic effects of *f* and *γ*(*D*).

## Graphical abstract

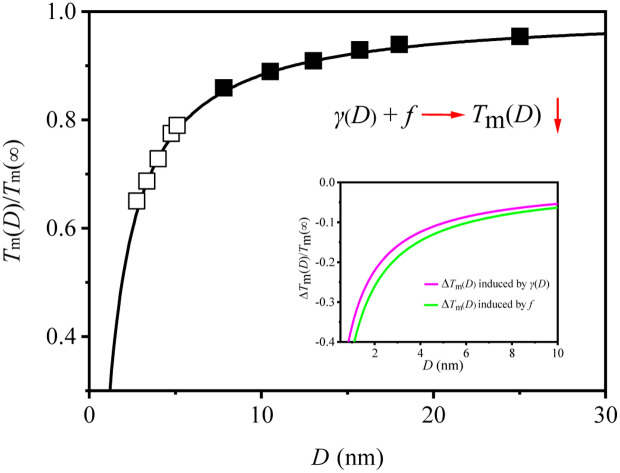

## Introduction

As a fundamental property in practical applications, the melting temperature *T*_m_ holds significant importance.^1–4^ Some useful material properties are functions of the ratio of working temperature (*T*_w_) to *T*_m_.^5,6^ Usually, for bulk materials, these functions become irrelevant due to the constant *T*_m_(∞), where ∞ denotes the bulk. Nevertheless, as the size (*D*) of metallic nanocrystals (MNCs) decreases into the nanoscale range, their melting temperature *T*_m_(*D*) also decreases due to the significant increase in surface-to-volume ratio (*A*/*V*).^7–9^ Simultaneously, *T*_w_/*T*_m_(*D*) increases with decreasing *D* and associated functions start to come into play. Consequently, at equivalent *T*_w_ values, MNCs can exhibit superior properties compared to bulk materials, which is advantageous for practical applications.^10,11^ Furthermore, the decrease in *T*_m_(*D*) will be a critical factor contributing to failure of many MNCs that operate at elevated temperatures.^12^ For efficient applications of MNCs, we should consider the trade-off between stability and properties to determine the optimal size at which both coexist. Additionally, certain properties of MNCs are directly or indirectly related to *T*_m_(*D*).^13,14^ Once we know how *T*_m_(*D*) behaves, it can serve as a bridge towards determining those size-dependent properties. Therefore, investigating the melting behavior of MNCs is crucial for determining the optimal size as well as identifying other size-dependent properties.

In order to understand the melting behavior of MNCs, extensive research has been conducted in both experimental and simulation fields. For example, electron diffraction patterns were used to analyze the thermostability of Pb nanoparticles (NPs).^15^ Additionally, the molecular dynamics method was utilized to study the melting behaviors of Pb nanowires (NWs)^16^ and Cu_*x*_Ni_1−*x*_ nanoalloys (NAs),^17^ with *x* representing the composition. In summary, the aforementioned references suggest that *T*_m_(*x*, *D*, *d*) can be customized by varying *D*, dimensionality (*d*) and *x*. To enhance our understanding of the melting behavior of MNCs and facilitate the design of nanodevices, it is imperative to comprehend the impact of these parameters.

Apart from experiments and simulations, theoretical studies are also effective in illustrating the melting mechanism. Many theoretical works have been conducted, such as Wautelet's Gibbs free energy model (FEM)^18^ and Nanda's Liquid-drop model (LDM).^19^ These two models share a common feature: *T*_m_(*D*) is inversely proportional to *D*, which can be expressed as:1
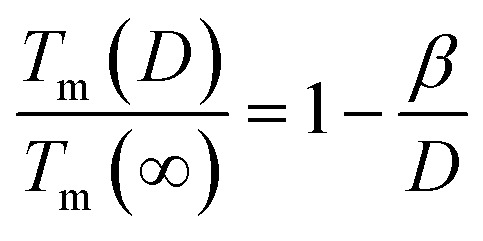


Although *β* is defined as a material constant, it has no explicit physical meaning and its value depends on the models employed. For instance, in the case of Pb, *β* can be 1.4 nm (ref. 18) in FEM or 1.8 nm (ref. 19) in LDM. In deriving these models, surface energy *γ* – an important thermodynamic parameter – was taken into account to determine the value of *β*. However, it should be noted that *γ* is size-dependent,^20,21^ whereas both LDM and FEM models treated it as a size-independent parameter, resulting in inaccurate values for *β*. Additionally, the reorganization of surface atoms when a new surface is formed can create surface stress. This surface stress, denoted as *f*, can result in surface strain and ultimately impact the *T*_m_(*x*, *D*, *d*) function.^21–24^ Specifically, both *γ*(*D*) and *f* can affect the *T*_m_(*x*, *D*, *d*) function. Therefore, to achieve a comprehensive understanding of the melting behavior of MNCs, it is essential to incorporate both factors into the model. In this contribution, the objective is to propose an analytical model that incorporates both *γ*(*D*) and *f* to predict the *T*_m_(*x*, *D*, *d*) function of MNCs. The accuracy and universality of our proposed model will be validated through comparison with corresponding experimental and simulation data.

## Model

As previously noted in our research, the role of cohesive energy per atom (*E*_c_) plays an equivalent role to that of *T*_m_ in determining thermostability,^25^ Specifically, *T*_m_(∞) is proportional to *E*_c_(∞). The absence of structural change should result in *T*_m_ and *E*_c_ being continuous functions as size decreases from bulk to nanoscale, with *A*/*V* increasing as the sole variable.^25^ Consequently, this relationship can be extended to the nanoscale level:2
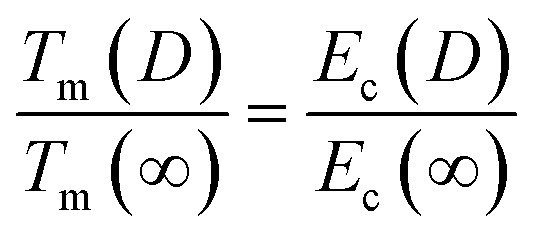


Therefore, the determination of *T*_m_(*D*) relies on knowledge of the *E*_c_(*D*) function. Compared with bulk crystals, MNCs possess a larger surface area, leading to the breaking of bonds of surface atoms and subsequent reorganization. The total energy of MNCs can be divided into three components: internal energy *E*_c_(*D*), surface free energy *γ*(*D*) arising from new surface formation, and distortion energy *U* caused by reorganization of surface atoms. According to the law of the conservation of energy, it can be expressed as:

3 *nE*_c_(∞) = *nE*_c_(*D*) + *Aγ*(*D*) + *U*

where *n* represents the total atoms number of MNCs.

For crystals with varying structures, the atoms arrange themselves in distinct patterns, resulting in diverse packing densities *η*. In the case of MNCs with a given volume *V*, *n* can be mathematically expressed as:4
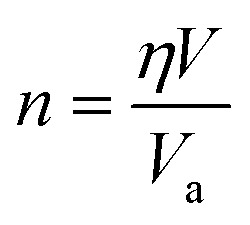
where *V*_a_ is the atomic volume.

According to our previous model and Qi's work, the approximate relationship between *γ* and *E*_c_ has been assessed as 
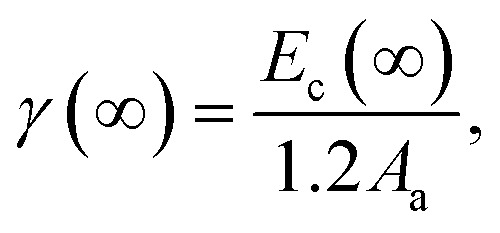
^26,27^ where *A*_a_ is the surface area of one atom. By extending this expression to the nanoscale, it can be reformulated as:5
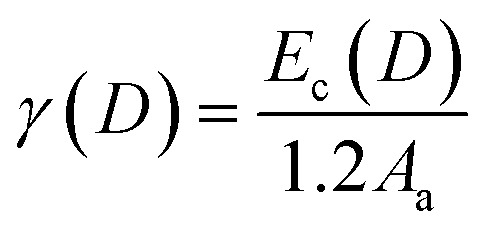
In eqn (3), *U* is another parameter that needs to be discussed. As mentioned above, *U* originates from the reorganization of surface atoms, resulting in the surface strain and imposing an additional internal pressure *P*_in_ at the surface. The distortion energy can be written as *U* = *P*_in_*V*.^28^ In accordance with the Laplace–Young equation, *P*_in_ is expressed as *P*_in_ = 2*Af*/3*V*, where *A*/*V* = 2(3 − *d*)/*D* and *d* takes values of 0, 1, 2 for NPs, NWs and nanofilms (NFs), respectively.^29^ Furthermore, the expression for *f* has been derived in Jiang's work:^30,31^

6 *f* = [(3*σ*_sl0_*D*_0_*B*)/8]^1/2^

where *D*_0_ represents the critical diameter and can be represented by *D*_0_ = 2(3 − *d*)*h*.^32^*B* denotes the bulk modulus. *σ*_sl0_ indicates the solid–liquid interface energy and has been deduced as follows:^33^

7 *σ*_sl0_ = 2*hH*_m_(∞)*S*_vib_(∞)/[3*V*_m_(∞)*R*]

where *h* is the nearest neighbor atomic distance, *H*_m_(∞) and *S*_vib_(∞) represent the bulk melting enthalpy and vibration entropy, respectively, while *V*_m_(∞) and *R* represent the molar volume and ideal gas constant. By substituting eqn (6) and (7) into the expression of *U*, we can derive:8
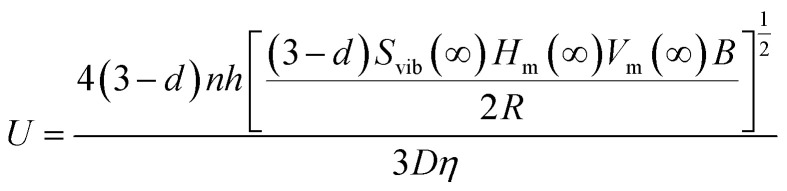


By combining eqn (3)–(5) and (8), *T*_m_(*D*, *d*) or *E*_c_(*D*, *d*) can be written as:9
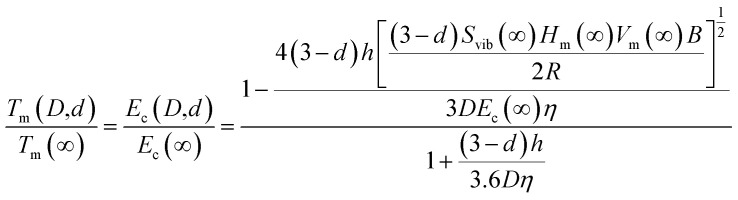


For NAs, the parameters required for calculation in eqn (9) are composition-dependent due to component interactions and can be approximated using the Fox equation:^34^10
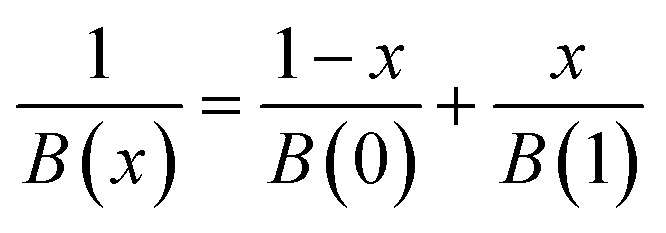
where *B* denotes parameters used in eqn (9).

## Results and discussion

**Fig. 1:**
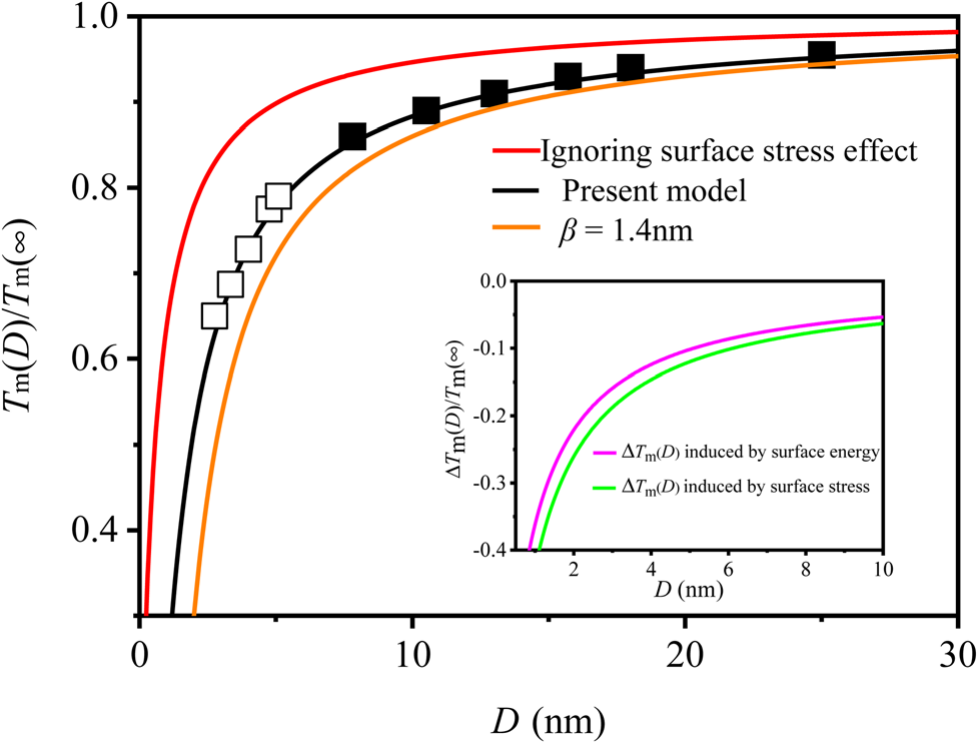
*T*_m_(*D*) function of Pb NPs. The symbols ■^15^ and □^35^ are the corresponding experimental and simulation data. The illustration expounds on the influences of *γ*(*D*) and *f* on the Δ*T*_m_(*D*).

Fig. 1 shows the model's prediction of *T*_m_(*D*) for Pb NPs based on eqn (9), represented by the black line. The parameters utilized in eqn (9) are detailed in Table 1. The general trend is that *T*_m_(*D*) decreases as *D* decreases, with a significant decrease occurring at *D* < 10 nm, indicating that size has a noticeable effect on *T*_m_(*D*) at smaller sizes. This tendency can be attributed to an increase in *A*/*V*. As the decline of *D*, a multitude of atoms attach to the surface, leading to an increase in *A*/*V*. Subsequently, the surface atoms take precedence in determining the properties of MNCs. Given that surface atoms possess higher energetic states than inner atoms, thermal vibration of MNCs intensifies as *D* declines. Based on this analysis and the Lindemann criterion, it is reasonable to anticipate a reduction in *T*_m_(*D*). During derivation of eqn (9), this decrease in *T*_m_(*D*) is attributed to synergistic effects between *f* and *γ*(*D*), however, existing analytical models scarcely consider the effect of *f* on *T*_m_(*D*). To determine whether *f* affects the *T*_m_(*D*) or not, we also plotted the prediction of ignoring the effect of *f* in eqn (9) as a red line in Fig. 1. As shown in Fig. 1, the red line is higher than both the present model prediction and the experimental (■^15^) and simulation (□^35^) data. However, good consistency can be observed between our present model prediction and these symbols. Therefore, it can be concluded that the existence of *f* has a decreasing effect on *T*_m_(*D*). From the perspective of energy, the presence of *f* can enhance the free energy of MNCs and subsequently decrease the *T*_m_(*D*). Additionally, Lu's research has demonstrated that *f* effectively reduces *T*_m_(*D*),^21^ thereby confirming the aforementioned analysis. Consequently, it is imperative to consider the impact of *f* as disregarding its effect may lead to an overestimation of *T*_m_(*D*).

**Table 1:** The relevant parameters are used in the calculation of the present model^a^

	*h*	*H*_m_(∞)	*T*_m_(∞)	*S*_vib_(∞)	*V*_m_(∞)	*B*	*E*_c_(∞)	*η* ^44^
Pb	0.350	4.77	600.6	6.65	18.26	46.00	2.03	0.74
Au	0.270	12.50	1337.3	7.62	10.21	220.00	3.82	0.74
Sn	0.290	7.00	505.1	9.22	16.29	58.00	3.06	0.53
Al	0.250	10.70	933.5	6.15	10.00	76.00	3.40	0.74
Bi	0.320	10.90	544.7	7.20	21.31	31.00	2.18	0.44
Ag	0.289	11.30	1234.9	7.82	21.31	100.00	2.94	0.74
In	0.368	3.26	429.8	7.58	15.76	43.00	2.52	0.68
Cu	0.270	13.10	1357.8	7.85	7.11	140.00	3.48	—
Ni	0.270	17.20	1728.0	8.11	6.59	180.00	4.47	—
Pd	0.275	16.70	1828.1	7.22	8.56	121.00	3.90	—
Pt	0.270	20.00	2041.4	7.80	9.09	230.00	5.85	—
Cu_0.2_Ni_0.8_^b^	0.270	16.20	1677.1	8.05	6.69	170.27	4.23	0.74
Cu_0.5_Ni_0.5_^b^	0.270	14.87	1520.7	7.98	6.84	157.50	3.92	0.74
Cu_0.8_Ni_0.2_^b^	0.270	13.76	1418.6	7.90	7.00	146.51	3.64	0.74
Pd_0.25_Ni_0.75_^b^	0.255	17.07	1752.0	7.87	6.99	160.44	4.55	0.74
Pd_0.5_Pt_0.5_^b^	0.272	17.44	1928.8	7.49	8.82	158.58	4.68	0.74
Pd_0.5_Cu_0.5_^b^	0.265	14.68	1558.2	7.52	7.77	128.33	3.69	0.74
Pb_0.5_Bi_0.5_^b^	0.335	6.64	571.1	6.92	19.67	37.00	4.72	0.74

a The units for *B*, *h*, *H*_m_(∞), *T*_m_(∞), *S*_vib_(∞), *V*_m_(∞) and *E*_c_(∞) are GPa, nm, kJmol^−1^, K, cm^3^ mol^−1^, J mol^−1^ K^−1^ and eV, respectively. The values of *B*, *h*, *H*_m_(∞), *T*_m_(∞) and *V*_m_(∞) are taken from the reference,^45^ the values of *S*_vib_ and *E*_c_(∞) are taken from the references.^46,47^.

b The parameters are calculated by eqn (11).

In addition, the prediction of FEM is also shown in Fig. 1 with an orange line for comparison. It is evident that the prediction of FEM is lower than both the symbols and the prediction of present model. During the derivation of FEM, *γ* was considered size-independent.^18^ However, it actually decreases with the decrease of *D*.^14,36^ According to FEM's expression, *T*_m_ (*D*) declines as increasing *γ*.^18^ Therefore, neglecting the size effect on *γ*(*D*) would lead to an overestimation of the impact of surface energy and a subsequent underestimation of *T*_m_(*D*). In light of the above analysis, it is imperative to consider the effects of *f* and *γ*(*D*), as failing to do so could lead to either an underestimation or overestimation of the size effect.

Based on the aforementioned analysis, both *f* and *γ*(*D*) are essential factors in determining the *T*_m_(*D*) function. To comprehend the individual impact of *f* and *γ*(*D*), one can express the difference in melting temperature (Δ*T*_m_(*D*) = *T*_m_(*D*) − *T*_m_(∞)) as follows using eqn (9):11
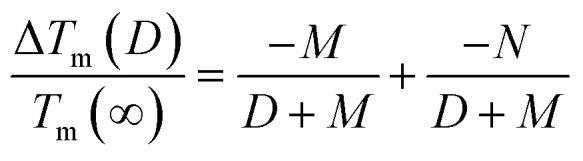
with 
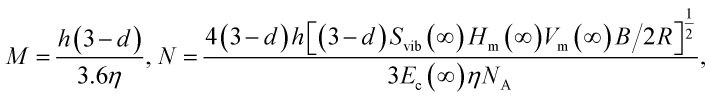
 denoting the influences of *γ*(*D*) and *f*, respectively. The influences on Δ*T*_m_(*D*) are depicted in the inserted graph of Fig. 1, where the green and pink lines represent their respective effects. As illustrated in the graph, both lines are below zero, suggesting that either *γ*(*D*) or *f* contributes to the decrease in *T*_m_(*D*). Additionally, the green line shows a more significant decline than the pink line, suggesting that *f* causes a greater depression in *T*_m_(*D*) depression compared to *γ*(*D*), as verified by Jiang's work.^37^

**Fig. 2:**
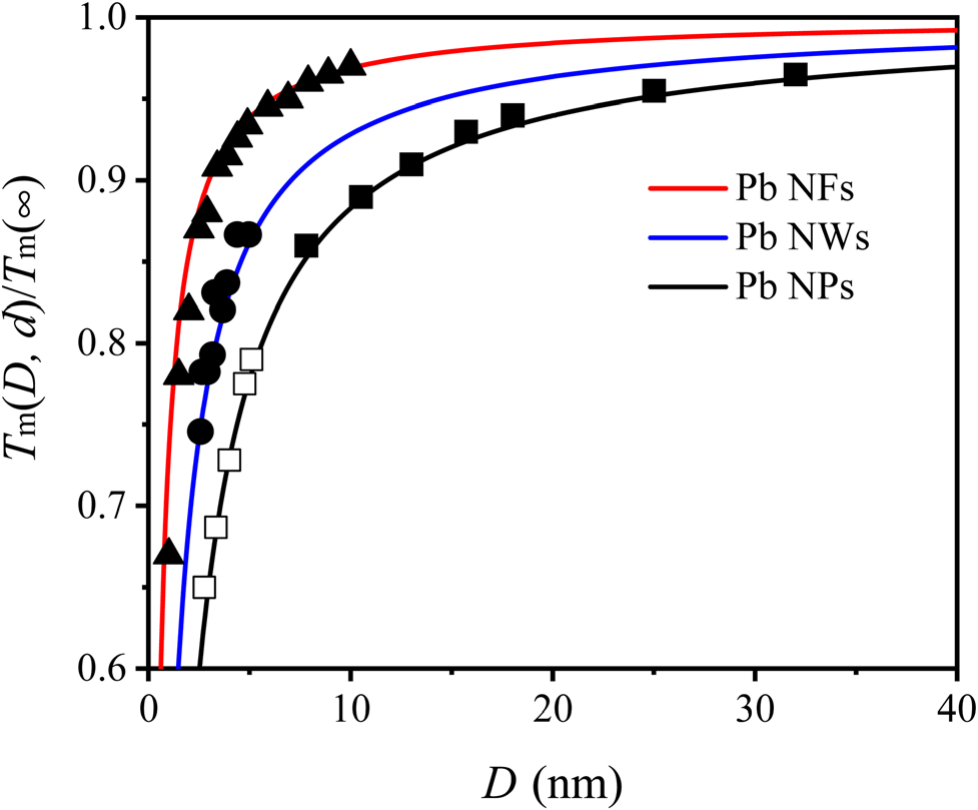
*T*_m_(*D*, *d*) functions of Pb MNCs. The symbols ▲^48^ and ●^16^ are the simulation data for Pb NFs, NWs, ■^15^ and □^35^ are the experimental and simulation data for Pb NPs, respectively.

Fig. 2 depicts the *T*_m_(*D*, *d*) function of Pb MNCs in light of eqn (9). The red, black and blue lines represent Pb NFs, NWs and NPs respectively from top to bottom. It is evident that all three lines decrease as *D* decreases. Furthermore, for a given *D* value, the *T*_m_(*D*, *d*) values can be ranked in descending order as follows: *T*_m_(*D*, 2) > *T*_m_(*D*, 1) > *T*_m_(*D*, 0). For MNCs with identical *D* but different *d* values, *A*/*V* can be sequenced as: (*A*/*V*)_NFs_ < (*A*/*V*)_NWs_ < (*A*/*V*)_NPs_. Based on the aforementioned analysis, the depression of *T*_m_(*D*, *d*) is caused by the increase in *A*/*V*. Henceforth, in terms of size effect, NPs exhibit the most significant impact followed by NWs while NFs exert a relatively weaker influence; this means that as *d* decreases, *T*_m_(*D*, *d*) also declines.

**Fig. 3:**
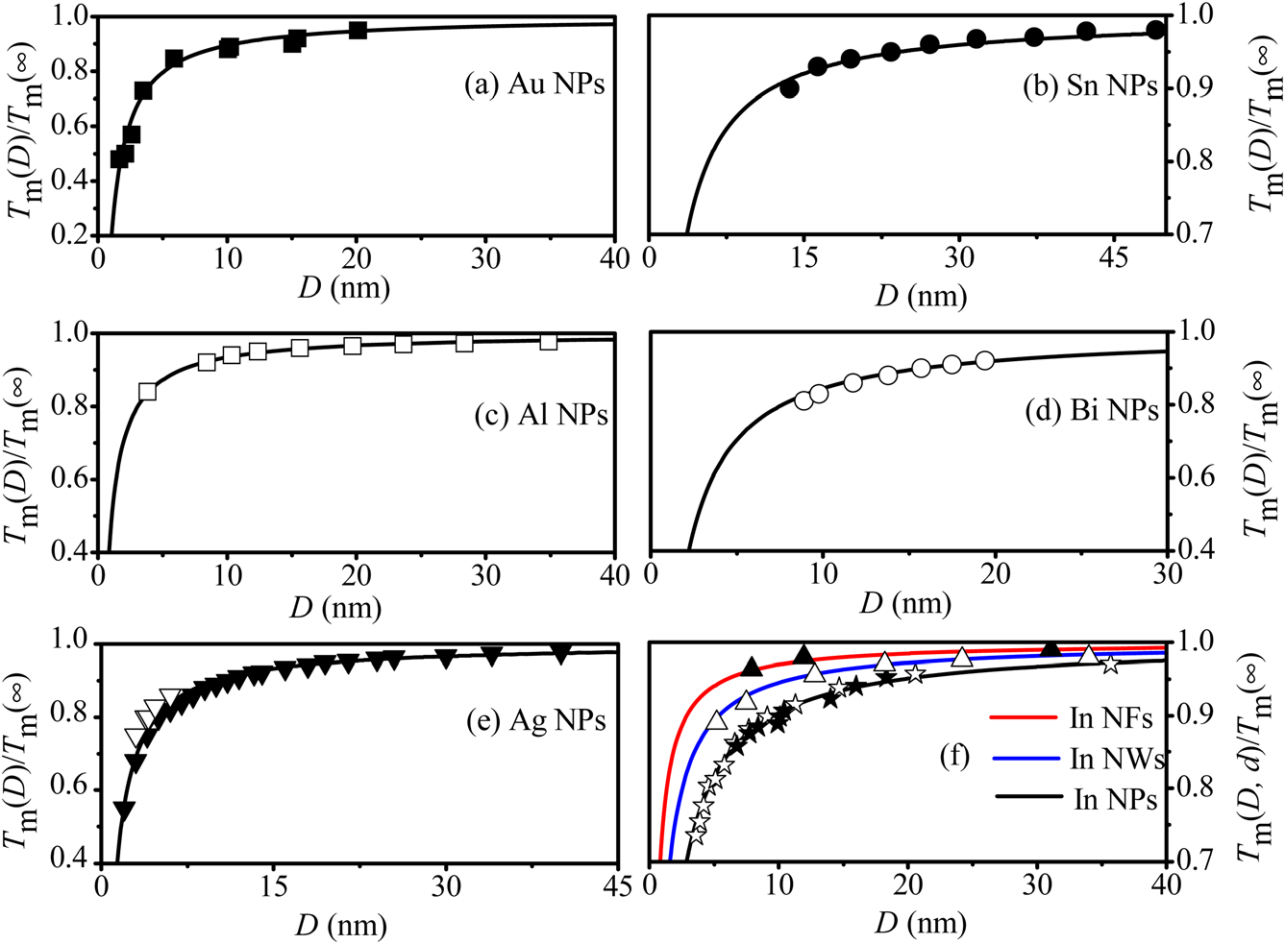
*T*_m_(*D*, *d*) functions of (a) Au NPs, (b) Sn NPs, (c) Al NPs, (d) Bi NPs, (e) Ag NPs and (f) in MNCs. The symbols ■,^49^ ●,^50^ □,^51^ ○^52^ ☆,^53^ ★,^54^ △^55^ and ▲^56^ are the experimental results, ▼^57^ and ▽^58^ are the simulation data.

To further demonstrate the universality of eqn (9), Fig. 3 presents a comparison between our model's predictions and experimental/simulation data for various MNCs, including Au, Ag, Al, Sn, Bi NPs and In MNCs. It is evident that the *T*_m_(*D*, *d*) curves of above MNCs are in agreement with their corresponding symbols. By combining Fig. 1–3, the validity and universality of the eqn (9) are fully demonstrated due to the remarkable consistency with both experimental and simulation data.

**Fig. 4:**
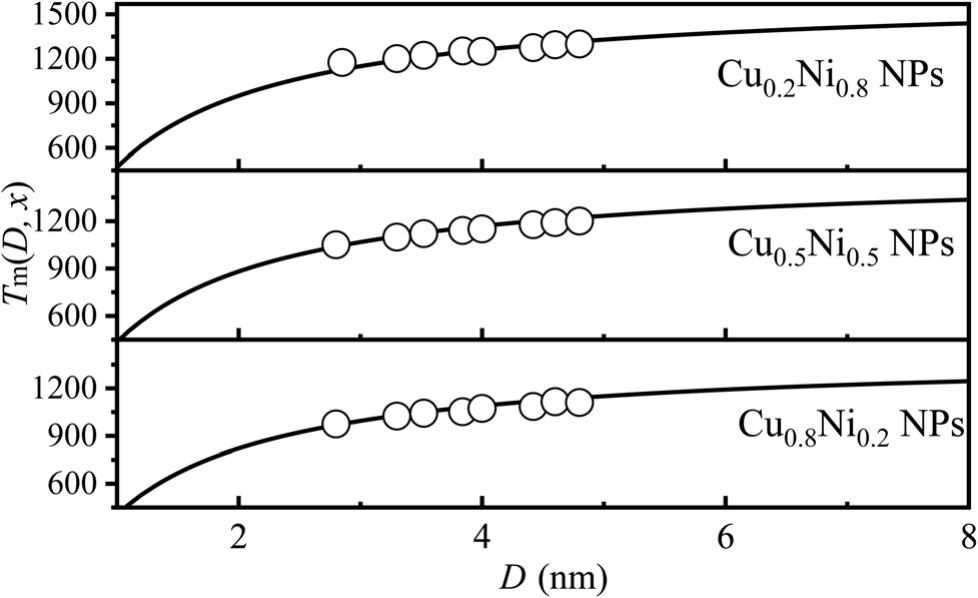
*T*_m_(*x*, *D*) functions of Cu_*x*_Ni_1−*x*_ NPs with *x* = 0.2, 0.5 and 0.8. The symbol ■^17^ is the simulation data.

For bimetallic NAs, the variation in component *x* can alter the interatomic interaction and subsequently impact melting temperature.^38^Fig. 4 illustrates the comparison between model predictions (Eq. (9)) and simulation results for *T*_m_ (*x*, *D*) of Cu_*x*_Ni_1−*x*_ NPs with *x* = 0.2, 0.5 and 0.8. It is evident that both of them exhibit a high degree of consistency. For instance, when *D* equals 4 nm, the simulation results are recorded at temperatures of 1250 K, 1150 K, and 1073 K,^17^ while the corresponding predicted values are 1260 K, 1167 K, and 1089 K for *x* equal to 0.2, 0.5, and 0.8, respectively. The margin of error between the two sets of data is approximately 1%. Furthermore, it also can be observed that, as Cu content increases, *T*_m_ (*x*, *D*) decreases. For NAs, the distribution of components is non-uniform, exhibiting surface segregation. The segregation phenomenon can be described as follows: the component with the lowest *γ* value preferentially segregates to the surface.^39,40^ Consequently, for the Cu_*x*_Ni_1−*x*_ NAs, Cu atoms will preferentially segregate to the surface due to their lower surface energy compared to Ni (*γ*_Cu_ = 1.566 J m^−2^*vs. γ*_Ni_ = 2.080 J m^−2^).^41^ With the increase of Cu, more surface segregation of Cu atoms leads to a decrease in the bond energy of Cu–Ni NAs, especially for surface atoms, and then leads to an increase in the thermal vibration of surface atoms. According to the Lindemann criterion,^42^ the decrease in *T*_m_(*x*, *D*) can be expected with the increase of the Cu component. The alignment between model predictions and simulation data confirmed the effectiveness of eqn (9).

**Fig. 5:**
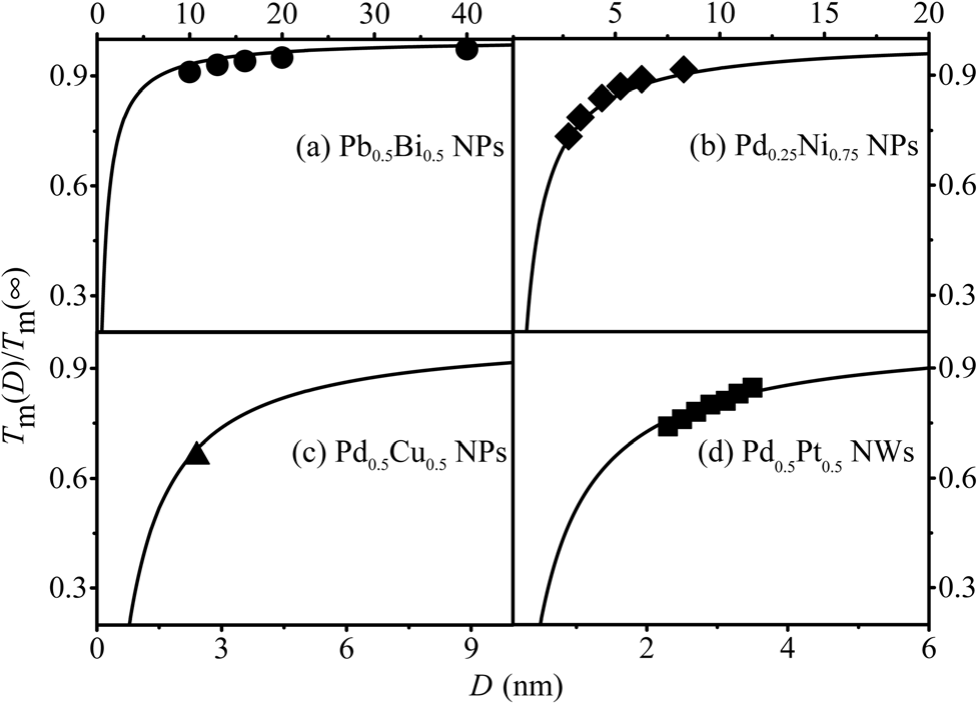
*T*_m_(*x*, *D*, *d*) functions of (a) Pb_0.5_Bi_0.5_ NPs, (b) Pd_0.25_Ni_0.75_ NPs, (c) Pd_0.5_Cu_0.5_ NPs and (d) Pd_0.5_Pt_0.5_ NWs. The symbol ●^59^ denotes the experimental results. ♦,^60^ ▲^61^ and ■^62^ are the corresponding simulation data, respectively.

Furthermore, the *T*_m_(*x*, *D*, *d*) functions for Pd_0.25_Ni_0.75_, Pd_0.5_Cu_0.5_, Pb_0.5_Bi_0.5_ NPs and Pd_0.5_Pt_0.5_ NWs are depicted in Fig. 5 to demonstrate the validity and universality of eqn (9), which is supported by both experimental and simulation data.

The model introduced in this study offers valuable insights into modifying *T*_m_(*x*, *D*, *d*) in MNCs by manipulating their size, dimensionality and composition. The parameters required for model prediction possess clear physical significance, which ensures the model's applicability not only to metallic nanocrystals but also to non-metallic nanocrystals. Given the critical importance of *T*_m_(*x*, *D*, *d*) in characterizing fundamental property variations,^13,14^ this model facilitates a quantitative exploration of key challenges in MNCs ' research. It is noteworthy that the model's applicability is currently restricted to MNCs under ambient pressure conditions. While as the pressure increases, the intermolecular distance of most substances decreases, leading to more stable solid structures that require higher temperatures to melt. Consequently, the melting temperature generally increases with increasing pressure,^3,43^ demonstrating an inverse relationship with the size effect. Therefore, our subsequent research will focus on exploring the competition between size-induced undercooling and pressure-induced overheating.

## Conclusions

In summary, a novel model is established by taking into account both *f* and *γ*(*D*), which can be utilized to predict the *T*_m_(*x*, *D*, *d*) of MNCs. The depression of *T*_m_(*x*, *D*, *d*) with decreasing *D* or *d* results from the synergistic effects of *f* and *γ*(*D*). While both *f* and *γ*(*D*) contribute to the reduction in *T*_m_(*x*, *D*, *d*), the impact of *f* is more pronounced than that of *γ*(*D*). Neglecting either factor would lead to an underestimation or overestimation of *T*_m_(*x*, *D*, *d*). Furthermore, as the component with the lowest *γ* increases, surface segregation causes a decrease in NAs' *T*_m_(*x*, *D*, *d*). By comparing it to corresponding experimental and simulation data, the validity and universality of the newly constructed model are confirmed, providing guidance for designing and applying new nanodevices.

## Abbreviation

*A*/*V*: Surface-to-volume ratio
*A*_a_
: Surface area of one atom
*B*
: Bulk modulus
*D*
: Size
*d*
: Dimensionality
*D*_0_
: Critical diameter
*E*_c_
: Cohesive energy per atom
*f*
: Surface stress
FEM: Gibbs free energy model
*h*
: Nearest neighbor atomic distance
*H*_m_
: Melting enthalpy
LDM: Liquid-drop model
MNCs: Metallic nanocrystals
NAs: Nanoalloys
NFs: Nanofilms
NPs: Nanoparticles
NWs: Nanowires
*P*_in_
: Addition internal pressure
*R*
: Ideal gas constant
*σ*_sl0_
: Solid-liquid interface energy
*S*_vib_
: Vibration entropy
*T*_m_
: Melting temperature
*T*_w_
: Working temperature
*U*
: Distortion energy
*V*_m_
: Molar volume
*x*
: Composition
*γ*
: Surface energy
*η*
: Packing density
*β*
: Material constant
∞: Bulk

## Data availability

All data that support the findings of this study are included within the article.

## Conflicts of interest

There are no conflicts to declare.
